# P2Y14 receptor activation and neutrophil signaling: linking inflammation to systemic pathophysiology

**DOI:** 10.1007/s11302-025-10113-7

**Published:** 2026-01-15

**Authors:** Renan da Silva Ebone, Pedro Henrique Doleski, Matheus Henrique Jantsch, Rafaella Pereira da Silveira, Daniela Bitencourt Rosa Leal

**Affiliations:** 1 Department of Microbiology and Parasitology, Universidade Federal de Santa Maria, Santa Maria, Brazil; 2 Postgraduate Program in Toxicological Biochemistry, Center of Natural and Exact Sciences, Universidade Federal de Santa Maria, Santa Maria, Brazil; 3 Postgraduate Program in Pharmaceutical Sciences, Center of Health Sciences, Universidade Federal de Santa Maria, Santa Maria, Brazil

**Keywords:** Purinergic, Inflammation, UDP-glucose, Chemotaxis

## Abstract

Neutrophils are essential effector cells of the innate immune system, acting as the first line of defense against infection and tissue injury. Among the purinergic receptors expressed in these cells, P2Y14 has gained increasing attention in recent years for its role in modulating neutrophil recruitment and activation in inflammatory contexts. This receptor is activated mainly by uridine diphosphoglucose (UDP-glucose) and other UDP-sugars released during cellular stress or damage. Through the activation of G protein–coupled pathways, particularly via Gi/o and RhoA signaling, P2Y14 influences key neutrophil functions, including chemotaxis, cytoskeletal rearrangements, and oxidative responses. Despite its pro-inflammatory potential, and the increasing amount of literature data in recent years, P2Y14’s complete physiological and pathological roles remain underexplored. Literature data also highlight its involvement in diseases like glioblastoma and COVID-19, where, due to increased neutrophil infiltration, it exacerbates inflammation, tissue damage, and stress. Therefore, targeting P2Y14 may be a promising strategy to modulate neutrophil chemotaxis and mitigate unwanted harmful inflammatory responses. This review discusses the characteristics and signaling mechanisms of P2Y14 in neutrophils, as well as the relevant implications of this pathway for neutrophil function.

## Introduction

The purinergic receptor P2Y14 is a metabotropic receptor highly expressed in various tissues and immune cells. It is activated by uridine diphosphate (UDP) and derivatives, but mainly uridine diphosphoglucose (UDP-glucose) and other UDP-sugars, such as UDP-galactose, UDP-N-acetylglucosamine, and UDP-N-glucuronic acid [1]. Although research on P2Y14 has increased in recent years, the receptor is still underexplored and has been demonstrated to be relevant in a variety of inflammatory and immune responses.

After eosinophils, neutrophils are the peripheral blood cells with the highest expression of P2Y14 mRNA [2], being human isolated or derived from HL-60 cell line differentiation [3]. These innate immune cells are the first line of defense in the human organism and are implicated in neuroinflammatory diseases and cancer in general, contributing to malignant cells’ survival and growth [4]. In turn, P2Y14 emerges as a relevant biochemical target that is capable of modulating neutrophil recruitment in different conditions, which increases the receptor’s therapeutic potential.

However, P2Y14’s range of action is not fully comprehended. Given its role in metabolic, immune, and inflammatory responses already described in the literature, P2Y14 emerges as a key mediator in neutrophil function, with potential implications in pathological conditions. Therefore, this review aims to explore the functional, physiological, and pathological characteristics of P2Y14 receptor activity in neutrophils and its connections to inflammatory and systemic diseases.

## Neutrophil physiology

Polymorphonuclear leukocytes (PMNs) are cells of the innate immune system and are among the first cellular components to initiate immunological responses against antigens. Neutrophils are the main subtype of PMNs, accounting for approximately 70% of total leukocytes. These cells exhibit distinctive morphological features that differentiate them from other leukocytes, such as a segmented nucleus with connected lobes and the presence of cytoplasmic granules. Together with basophils and eosinophils, they form the group of granulocytes [5].

Neutrophils originate primarily from the bone marrow, derived from hematopoietic stem cells, and in normal health conditions are the most abundant leukocytes in the human bloodstream, with approximately one hundred billion cells produced daily [6, 7]. Despite their abundance, neutrophils have a short lifespan; after being activated and migrating to damaged tissues, they survive for less than 2 days. Nevertheless, neutrophils can differentiate into various subsets with distinct functions, which may affect their lifespan and role in immune responses. This plasticity allows them to adapt to different pathological conditions, such as cancer and inflammation [8, 9].

The main functions neutrophils exert include phagocytosis, degranulation, NETosis (neutrophil extracellular trap synthesis), and cytokine release [7]. They can act as mediators in the production of antimicrobial peptides, such as defensins and cathelicidin, stored in specific cytoplasmic granules, and they can externalize their chromatin as a defense mechanism, forming NETs, where histones and DNA strands combine with granular contents to kill pathogens. However, this process leads to NETosis, the cell death of the neutrophil, a process implicated in several diseases due to its pro-inflammatory nature [10]. To engulf and eliminate infectious agents is a role performed not only by macrophages, but also by neutrophils, which gives them a phagocytic profile [11]. Once the antigen is inside the phagocyte, it is neutralized through the action of reactive oxygen species (ROS), nitrogen species, and proteases [12]. Phagocytic cells also generate responses against infectious agents and cellular damage by secreting inflammatory cytokines and chemokines that can further recruit different leukocytes to enhance the immune response against the antigens [13].

Neutrophils express several receptors of the purinergic system, including various P2X and P2Y, as well as all four P1 receptors (A1, A2A, A2B, and A3) [14]. Uridine nucleotides, such as UTP, play roles similar to those observed with ATP interaction in neutrophil activation, promoting chemotaxis and ROS production via P2Y2 [15]. Meanwhile, UDP acts in NET formation through P2Y6 [16]. Unlike adenosine and its derivatives, which can be hydrolyzed by regulatory ecto-enzymes, UDP-glucose and other UDP-sugars are not easily metabolized extracellularly and are considered more stable extracellular signals [17]. Therefore, when stimulated by UDP-glucose, neutrophil’s Rho GTPases are activated, playing a key role in cytoskeletal rearrangements that are necessary for cell motility and migration. These processes are mediated by P2Y14 activation, which is a crucial receptor for neutrophil’s chemotaxis [3, 18].

## P2Y14 background

Before P2Y14 was even assigned to the family of purinergic signaling receptors, it was formerly discovered as a human clone for a G-protein-coupled receptor (GPCR) [19]. GPCRs are a wide family of receptors that respond to a variety of extracellular stimuli, such as amino acids, inorganic acids, lipids, proteins, nucleotides, and even to photons. Many of these GPCRs were called orphan receptors for not having a natural ligand yet discovered, and among these orphans, there was one called KIAA0001, widely expressed in mammalian cells. Later, studies determined that KIAA0001 was responsive for UDP-glucose and other UDP-sugars acting as extracellular signals, indicating that besides their regular metabolic roles of glycosyl donation in the carbohydrate’s biosynthesis, such molecules could also act as important physiological mediators [20].

There were several similar characteristics between KIAA0001 (also renamed with other murine orthologues as GPR105) [21] and the purinergic receptors belonging to the P2Y subgroup. Abbracchio et al. (2003) compared these similarities and found preserved motifs in transmembrane regions of GPR105 that are crucial for nucleotides binding; the chromosomal region where GPR105 is found relates to other P2Y receptors, suggesting an evolutive relation between them; the activation of GPR105 by UDP-glucose is coupled to G proteins from the Gi/o class; and finally, the specific response to UDP-glucose (or UDP-galactose and other UDP-sugars) indicated that GPR105 was a unique receptor, differing from other P2Y receptors, suggesting that the studied receptor was not only structurally but also physiologically similar [19].

These findings allowed GPR105 to change its nomenclature by the International Union of Pharmacology (IUPHAR) to P2Y14, entering the purinergic system’s family (despite its curious activation by pyrimidine nucleotides rather than purines) [19]. Given the historical background, it is essential to explore the main characteristics of P2Y14.

## P2Y14 characteristics, localization, and functioning

**Fig. 1:**
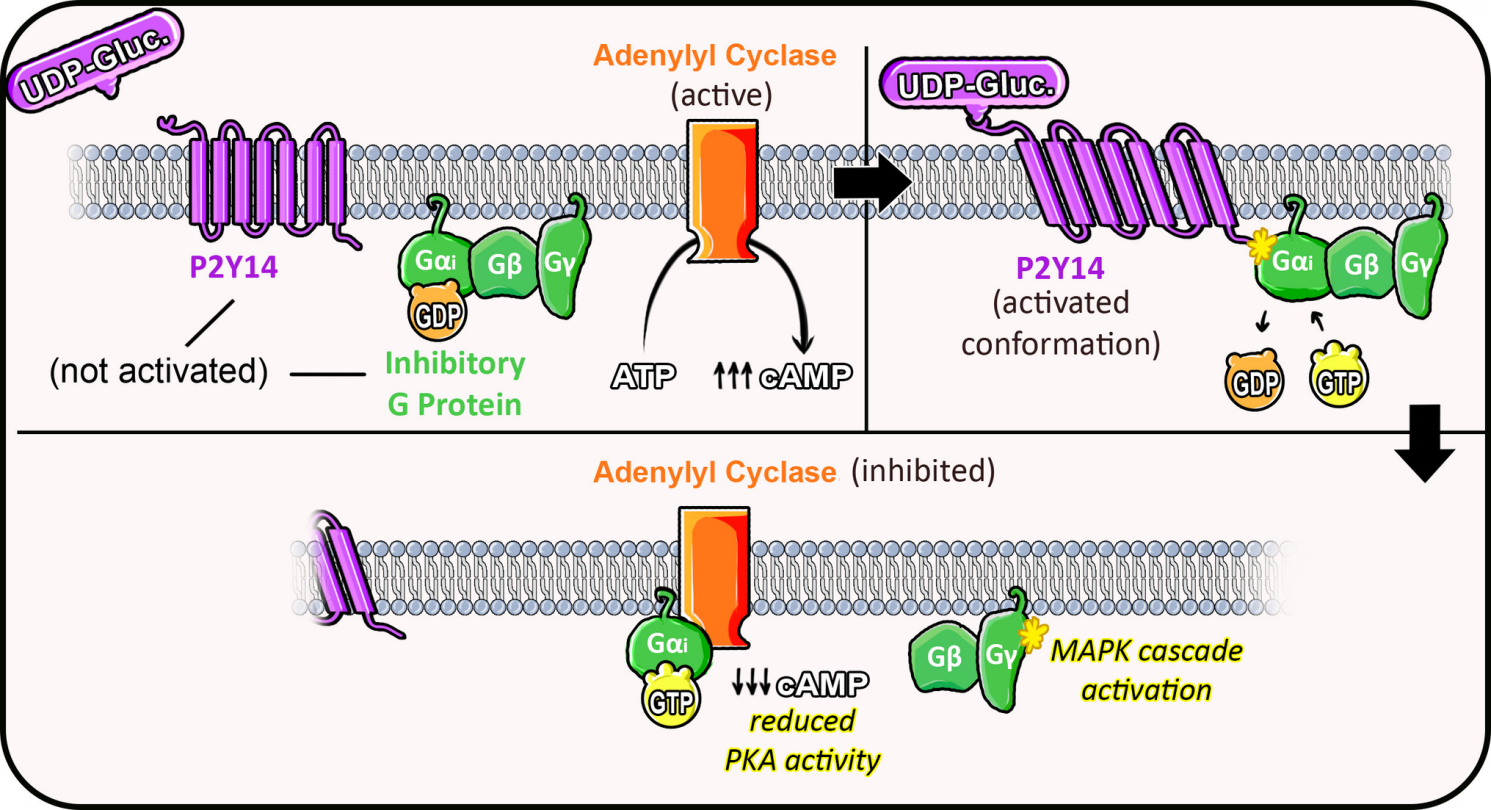
Basic mechanism of P2Y14 activation and G protein signaling. Upon binding of UDP-glucose to the P2Y14 receptor, a conformational change occurs, allowing interaction with the inactive heterotrimeric Gi protein. This interaction promotes the exchange of GDP for GTP on the Gαi subunit, leading to dissociation of the G protein into two active components: Gαi–GTP and the Gβγ dimer. The Gαi–GTP subunit inhibits adenylyl cyclase, reducing cAMP production by preventing ATP conversion. Concurrently, the Gβγ dimer can activate downstream effectors, including components of the MAPK signaling cascade. Together, these pathways modulate intracellular second messenger systems in response to extracellular UDP-glucose stimulation

Similar to other P2Y receptors, P2Y14 structure is composed of seven transmembrane domains [19], and the receptor’s roles can vary among different cell types. Like other GPCRs, P2Y14 interacts with an intracellular G protein that activates or inhibits specific signaling pathways depending on its structure. Therefore, this receptor is specifically coupled to the inhibitory G protein (Gi) [2], and as a GPCR, it triggers the dissociation from the inactive G protein form to the active G protein form, as depicted in Fig. 1. Through its coupling with Gi proteins, it is well known that P2Y14 activation by its agonist indirectly causes the inhibition of adenylyl cyclase through G protein αi subunit, thereby reducing cyclic AMP (cAMP) levels, altering the downstream cascade of intracellular signals in the cell [22], but it may also affect other protein cascades such as protein kinases, cell excitability by K channels, and phospholipase C activation through G protein βγ subunits influence [23].

In contrast with other purinergic receptors that are activated by classic nucleotides/nucleoside such as ATP, ADP, or adenosine, P2Y14 is curiously selective with its main agonists, which are molecules conjugated with sugars: uridine 5′-diphosphoglucose (UDP-glucose), UDP-galactose, UDP-N-acetylglucosamine, and UDP-N-glucuronic acid [20], besides UDP itself being a potent agonist for this receptor as well [22]. However, differently from adenine nucleotides that are quickly hydrolyzed by ectonucleotidases and apyrases, and unlike UDP that can be easily converted to UTP by nucleoside diphosphokinase (NDPK) [17], UDP-sugars are hydrolyzed by ecto-nucleotide pyrophosphatase/phosphodiesterases (E-NPPs)[24].* This enzymatic pathway confers* a greater molecular stability for UDP-sugars, allowing them to act as persistent pro-inflammatory signals interacting with this receptor, which is widely distributed in various cells, both human and rodent [20].

P2Y14 is expressed in a variety of tissues like the placenta, intestine, brain, kidney, uterus, epididymis, adipose tissue, and hepatic stellate cells. It can also be found in hematopoietic cells of the bone marrow, and in immune cells, such as mast cells, lymphocytes, macrophages, and neutrophils [25]. Notably, P2Y14 is also expressed on platelets, and recent studies demonstrate that the receptor participates in platelet migration, but not in platelet aggregation and neither thrombus formation [26]. Beyond these initial findings, the role of P2Y14 on platelets remains poorly defined.

However, considering the well-established contribution of platelets to neutrophil recruitment and activation, platelet P2Y14 may represent an additional regulatory component of platelet–neutrophil communication. Besides Hossain et al.’s (2024) studies, the literature data lack strong evidence of P2Y14 direct effects on platelets, in contrast to well-documented influence on other immunological cells, particularly neutrophils [27]. Nevertheless, we further discuss in the “P2Y14’s chemotactic effect on neutrophils” section the role of P2Y14 receptors expressed on platelets that may contribute to neutrophil migration and activity.

Beyond the canonical cAMP pathway of second messengers, intracellular Ca^2+^ influx has also been reported in the P2Y14’s mechanisms, evidenced in some studies. For example, the activation of the P2Y14 receptor by UDP-glucose in immature monocyte-derived dendritic cells (MDDCs) induces Ca^2+^ influx and increases CD86 expression (a co-stimulatory molecule in the antigen presentation to T cells) in some donors, but not in mature MDDCs or monocytes, suggesting that it plays a role in the maturation and in the activation of immature MDDC [28].

On astrocytes, the receptor was also demonstrated to increase platelet Ca^2+^ mobilization, an event that was prevented with the administration of P2Y14 siRNA to these cells, supporting the role of the receptor on transitory intracellular ion levels [29, 30]. The receptor also demonstrated an increase in platelet Ca^2+^ mobilization, an event that did not occur with the administration of the P2Y14 direct antagonist molecule, the PPTN (4-((piperindin-4-yl)-phenyl)-(7-(4-(trifluoromethyl)-phenyl)−2-naphthoic acid) [26]. PPTN is a high-affinity and selective antagonist of the P2Y14 that does not affect any of the other known P2Y receptor subtypes [31]. Nevertheless, P2Y14’s main roles are implicated in immune responses, particularly in the context of cell damage and the processes involved during inflammation.

## UDP-glucose’s inflammatory signaling via P2Y14

The P2Y14 receptor was pharmacologically characterized in HEK-293 cells transfected with P2Y14, which revealed half-maximal effective concentrations (EC_50_) of approximately 100 nM (0.1 µM) for UDP-glucose [20]. The sensitivity to UDP-glucose also varies among cell types, with values indicating that micromolar concentrations of this agonist are necessary to trigger P2Y14-mediated signaling events [32, 33]. The extracellular origin of UDP-glucose derives from secretion and passive diffusion in healthy cells within the nanomolar range, or from the release of intracellular content through membrane damage and cell death, raising UDP-glucose concentrations to micromolar levels [24, 34, 35].

Sesma et al. (2016) described that, in the bronchoalveolar lavage fluid (BALF) of healthy individuals, the low concentration of UDP-glucose (approximately 10 nM) is far below that required for P2Y14 activation. However, in the BALF of patients with cystic fibrosis, the concentration of UDP-glucose increases to above 100 nM (0.1 µM), thereby inducing effects via P2Y14 [36]. In assays assessing neutrophil chemotaxis and functional activation of downstream effectors such as RhoA, the reported EC_50_ for UDP-glucose generally ranges between 0.4 and 0.9 µM in primary neutrophils and neutrophil-like HL-60 cells [3, 25].

The degradation of extracellular UDP-glucose occurs mainly through the action of E-NPPs, which hydrolyze the pyrophosphate bond of the molecule, generating glucose-1-phosphate and uridine monophosphate (UMP). This pathway has been experimentally confirmed by the detection of glucose-1-phosphate as the main product of UDP-[^3^H]glucose metabolism (in which a hydrogen is substituted with tritium for radiolabeling). The half-life of UDP-glucose in the extracellular medium has been estimated at 77 min, approximately three times longer than that of ATP, which also explains its sustained presence following stimulation [24].

When a tissue is injured by stress or mechanical damage, the nucleotide/nucleoside signaling molecules (such as purines like adenosine and its derivatives, and pyrimidines like UDP and UDP-sugars) are released in the extracellular medium and act as damage-associated molecular patterns (DAMPs). These DAMPs promote sterile responses to the PRRs (pattern recognition receptors), like the receptors of the purinergic system, and trigger immune cells’ response to the damage [37]. P2Y14 is present in several tissues, and when these cells are injured, for instance, UDP-glucose is released to the extracellular medium and interacts with the receptor located on neighboring cells through a paracrine signaling mechanism [38]. This interaction activates Gi protein, causing the Gαi-GTP subunit to inhibit adenyl cyclase, affecting second messengers’ cascade [38], while the Gβγ dimer can interact with a variety of intracellular processes that vary from cell to cell, like the activation of MAPK cascade [39].

**Fig. 2:**
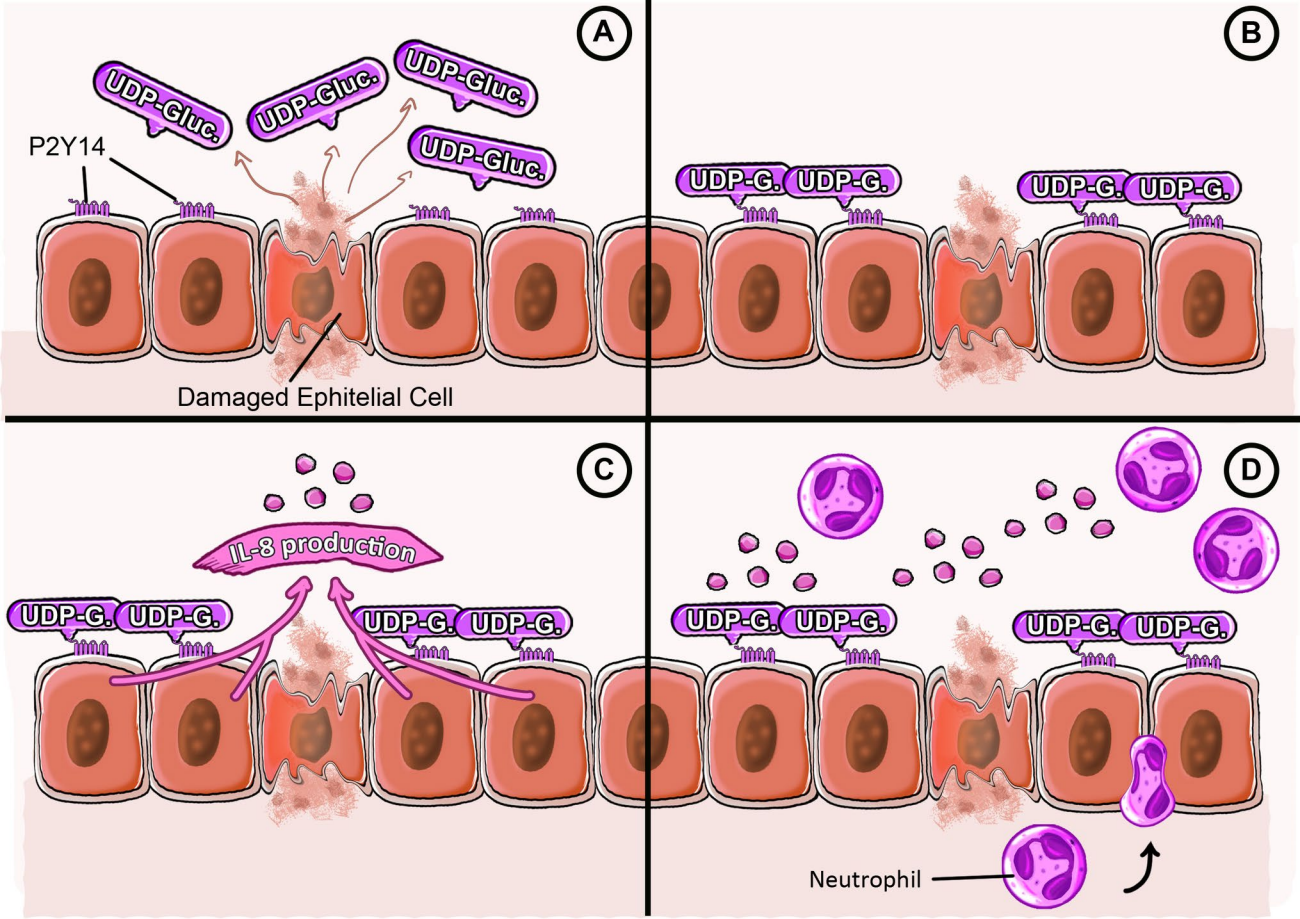
UDP-glucose release and P2Y14 binding. **A** Cellular damage leads to the release of intracellular components, including UDP-glucose, into the extracellular space. **B** Extracellular UDP-glucose binds to P2Y14 receptors expressed on neighboring epithelial cells. **C** P2Y14 activation triggers intracellular signaling cascades that culminate in the production and secretion of IL-8. **D** IL-8 acts as a chemoattractant, promoting the recruitment and directed migration of neutrophils toward the site of injury

These signaling events trigger the cell’s interleukin-8 (IL-8) production in humans, a strong chemotactic molecule for neutrophils, and functionally equivalent chemokines in murine species (like macrophage inflammatory protein-2 and keratinocyte-derived cytokine). Consequently, this metabotropic cascade promotes neutrophil recruitment and infiltration to the damaged site in a sterile manner [38], as depicted in Fig. 2. However, the elevation of extracellular UDP-glucose concentrations during pathological processes is also related to its active release through mechanisms such as vesiculation, anion channels, or calcium-dependent exocytosis, interacting with the receptor through autocrine and paracrine signaling mechanisms, rather than simple cell rupture [40].

The relatively low affinity of P2Y14 for UDP-glucose is compensated by the persistent presence of this agonist in the extracellular environment, which results from its long half-life and regulated release mechanisms. Together, its regulated release and EC_50_ values reinforce the role of UDP-glucose as an important modulator of the innate immune response at the micromolar level, such as in mast cell activation, neutrophil recruitment, and cytokine production [36].

## P2Y14’s chemotactic effect on neutrophils

In the context of leucocyte recruitment, P2Y14 exerts a relevant role during inflammation over different immune cells, like eosinophils and monocytes, even though its functioning is highlighted mainly on neutrophils [41]. Beyond chemotaxis, recent evidence demonstrates that UDP-glucose can also activate neutrophils through P2Y14, promoting respiratory burst and oxidative stress, as part of their effector response. This activation involves p38 MAPK [42] and RhoA (a Rho GTPase) signaling, both key regulators for neutrophil activity. RhoA increases the cell chemotaxis by cytoskeletal rearrangements and changes in the cell shape, facilitating motility and migration to the inflammatory site [3].

In basal conditions, however, P2Y14 seems not to be highly expressed on neutrophils, though they functionally express the receptor, but specific inflammatory stimuli can increase the receptor expression. For instance, cell injury or stress conditions are inflammatory events that stimulate the receptor expression in neutrophils [43]. As demonstrated by Li et al. (2023), after induced myocardial infarction, UDP-glucose released from damaged cardiomyocytes stimulates P2Y14 expression in heart neutrophils. This inflammatory event promotes other leukocytes’ recruitment and neutrophil migration to the injured tissue [43].

The chemotaxis effect of P2Y14 on neutrophil migration during inflammation can be noted in other tissues as well. For example, it was observed both in renal and lung cell line studies in vitro, where on dose-dependent concentrations of UDP-glucose, this nucleotide signaling increased the phosphorylation of the extracellular signal-regulated kinase 1 and 2 (ERK1/2). Consequently, UDP-glucose increased pro-inflammatory cytokine and chemokine release from these cells, which further promoted neutrophil recruitment. In airway epithelial cells, these effects were shown to depend on Gi protein signaling, since they were prevented by either the P2Y14 receptor antagonist PPTN or by pertussis toxin, which directly inhibits Gi protein activation and downstream signaling [44].

In contrast, in the kidney, P2Y14 receptor expression was mainly found in intercalated cells, where UDP-glucose stimulated ERK1/2 phosphorylation and chemokine expression both in vitro and in vivo. In these studies, blockade of P2Y14 with PPTN mitigated ERK1/2 activation and neutrophil recruitment induced by UDP-glucose [45]. More recently, a study combining pharmacological inhibition of P2Y14 with an intercalated cell–specific P2Y14 knockout mouse model demonstrated that P2Y14 signaling drives renal inflammation and neutrophil recruitment. In this model, several cytokines and chemokines, including CXCL1, CCL2, CXCL2, IL-1β, and IL-6, were elevated by injury induction, but were significantly attenuated by P2Y14 inhibition [46].

Similarly, in endometrial epithelial cells derived from humans, P2Y14 activation by UDP-glucose stimulated IL-8 production and further neutrophil recruitment. Arase et al. (2009) demonstrated that this event occurred also in the murine uterus administered with UDP-glucose, which released murine IL-8–like chemokines and increased leucocyte recruitment to the administered tissue, such as monocytes, but mainly neutrophils. When administered with P2Y14 siRNA, there was a reduction in neutrophil recruitment in this study, supporting the chemotactic effect of P2Y14 [38].

Regarding activated platelets, these cellular fragments promote neutrophil chemotaxis and activation through multiple pathways, including the exposure of adhesion molecules such as P-selectin and CD40L, and the release of soluble mediators like CXCL4, CCL5, and HMGB1. These signals stimulate neutrophil adhesion, activation, and NET formation, which collectively amplify thromboinflammatory responses [47]. Clinically, increased platelet–neutrophil aggregates have been associated with acute thromboinflammatory conditions such as symptomatic carotid stenosis [48], and changes in neutrophil-to-platelet ratios have been identified as predictors of poor outcomes in sepsis, further underscoring the importance of platelet–neutrophil crosstalk in inflammation and vascular injury [49].

More recently, Fang et al. (2025) provided evidence that P2Y14 signaling in neutrophils promotes venous thrombosis by enhancing platelet-induced NET formation through the PKA/AKAP13/RhoA pathway. Genetic or pharmacological inhibition of P2Y14 in neutrophils reduced platelet–neutrophil complex formation and alleviated thrombus burden, revealing that P2Y14 directly influences neutrophil–platelet interactions and thromboinflammatory progression [50]. Although these effects were attributed primarily to neutrophil P2Y14, they highlight a close mechanistic interplay between the two cell types that may also involve platelet P2Y14.

Indeed, Hossain et al. (2024) demonstrated that platelet P2Y14 activation triggers Ca^2+^ mobilization and Rho-GTPase-dependent cytoskeletal remodeling through Gi-coupled signaling and interaction with P2Y1 receptors, promoting platelet motility without inducing aggregation or leukocyte binding [26]. Such cytoskeletal activity could enhance platelet positioning and paracrine communication within inflamed or thrombotic microenvironments, indirectly favoring neutrophil recruitment [26, 47, 50].

Therefore, a cooperative mechanism involving both platelet and neutrophil P2Y14 receptors could exist, potentially amplifying chemotactic and thromboinflammatory responses. This hypothesis aligns with the demonstrated role of P2Y14 in cytoskeletal regulation and chemotaxis, but requires experimental confirmation to clarify how this signaling axis integrates platelet and neutrophil functions in the pathophysiology of immunothrombosis.

## P2Y14 and the G protein pathways

When it comes to P2Y14 activation, the adenylyl cyclase inhibition mediated by the Gαi subunit is the most classical characteristic. However, the Gβγ dimer is demonstrated to be involved in the cellular processes directly linked to P2Y14 effects reported in the literature. Nevertheless, the activated G protein subunits’ intracellular interactions directly through P2Y14 activation remain not fully described in the literature data due to their complexity.

As discussed in the “UDP-glucose’s inflammatory signaling via P2Y14” section, the P2Y14 interaction with UDP-glucose, as a GPCR, may lead to MAPK cascade activation, a correlation also observed by Lazarowski and Harden (2015) [23]. One proposed mechanism for this outcome involves Gβγ dimer’s well-documented interaction with phosphoinositide 3-Kinase (PI3K) on the membrane [51]. In overall GPCR signaling, the Gβγ dimer has also been proposed to translocate to the Golgi apparatus, where it may activate ERK1/2 proteins (key factors for cell functioning) via a PI3K-dependent mechanism, as detailed by Kather, Bryant, and Wu (2021). However, this mechanism has not been directly demonstrated for P2Y14 [52].

**Fig. 3:**
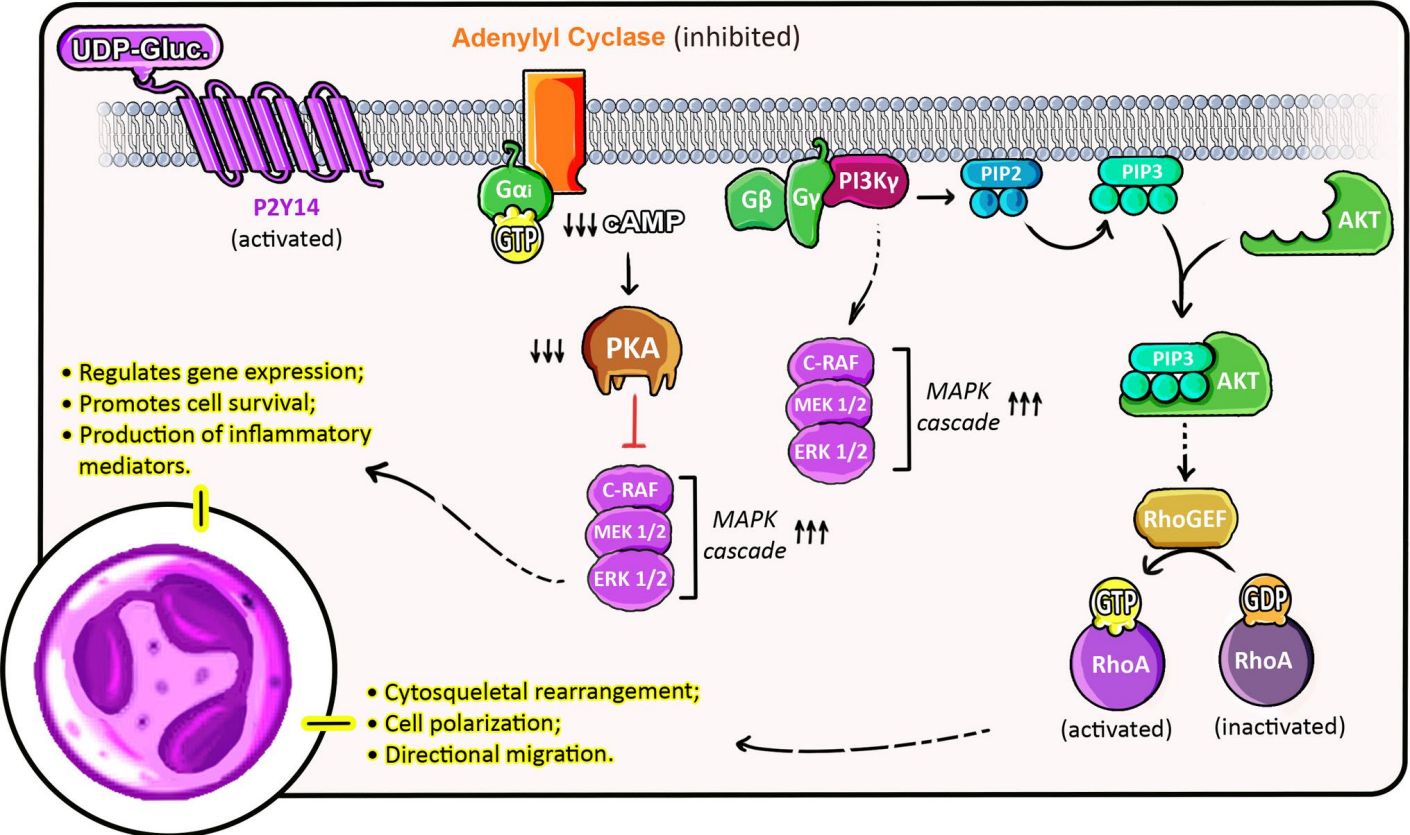
Proposed P2Y14 signaling pathways in neutrophils. Upon activation of the heterotrimeric Gi protein by P2Y14, the Gαi subunit inhibits adenylyl cyclase, leading to reduced cAMP levels and suppression of PKA activity. This is hypothesized to facilitate the activation of MAPK cascade, including ERK 1/2, that enhances nuclear functions. Concomitantly, Gβγ is proposed to activate PI3K, catalyzing the conversion of PIP2 to PIP3. PIP3 recruits and activates AKT, which in turn possibly stimulates the recruitment of RhoGEFs that convert inactive RhoA-GDP into active RhoA-GTP form. Finally, RhoA enables front edge dynamics necessary for migratory capacity. Together, these signaling branches regulate key effector functions of neutrophils upon UDP-glucose binding to P2Y14. The activation of RhoA has been directly demonstrated in neutrophils, while the PI3K/AKT/RhoGEF interactions are proposed based on general GPCR signaling

P2Y14 activation has been demonstrated to have a correlation with Rho GTPases’ activity. Sesma et al. (2012) described that P2Y14 activation by UDP-glucose and the other UDP-sugars promoted RhoA activation along with cytoskeletal rearrangement in neutrophils, but not in neutrophils lacking P2Y14 mRNA [3]. However, the direct link between P2Y14 and PI3Kγ activation remains to be fully elucidated, although it is plausible given the well-known role of Gβγ in PI3Kγ activation in neutrophils [51]. It is known that PI3K phosphorylates phosphatidylinositol 4,5-bisphosphate (PIP2) into phosphatidylinositol 3,4,5-trisphosphate (PIP3), which then binds to AKT. PIP3/AKT has been reported to participate in the activation of Rho GTPase pathways, which are key regulators of cytoskeletal dynamics and cell migration, while also facilitating the recruitment of Rho GEFs (guanine exchange factors) to the plasma membrane. This interaction with Rho GEFs enhances Rho GTPase activity, promoting cell adhesion, cytoskeletal rearrangement, and motility [53], as depicted in Fig. 3.

PIP3’s crucial role in neutrophil chemotaxis has also been demonstrated by its accumulation at the leading edge of the cell, creating a positive feedback loop that reinforces Rho GTPase recruitment and improves cell polarization. When PIP3 was inhibited in vitro, neutrophils’ movement became erratic, supporting the role PIP3 plays in maintaining the leading edge for neutrophil directional movement toward the chemoattractant molecule’s gradient, such as IL-8 [54]. Thus, PI3K plays a critical role in neutrophil chemotaxis pathways [55], which may be driven by Gβγ activity through G protein activation. These characteristics suggest that UDP-glucose binding to P2Y14 promotes similar downstream effects, including PI3Kγ activation and RhoA, which mediates cytoskeletal rearrangement in neutrophils [56]. Sesma et al. (2012) demonstrated PI3K and RhoA activation upon P2Y14 stimulation [3], although the full pathway remains to be demonstrated.

## P2Y14 beyond neutrophils

Several studies have demonstrated that P2Y14 plays a significant role in various other inflammatory and pathological contexts, not only those of neutrophilic nature. In acute gouty arthritis, Li et al. (2020) showed that P2Y14 upregulates macrophage pyroptosis by inhibiting cAMP production, which is crucial for the ubiquitination and degradation of the inflammasome. Therefore, activation of this receptor enhances inflammasome assembly on macrophages and promotes excessive cytokine release, exacerbating inflammation. Conversely, its inhibition attenuates NLRP3 inflammasome formation and reduces cytokine secretion, attenuating the inflammatory role that the receptor exerts in this context [57].

UDP-glucose is also abundantly present in inflamed intestinal tissues, such as observed in ulcerative colitis. Acting as a DAMP that recruits eosinophils, the interaction among this agonist and P2Y14 activates the ERK1/2 signaling pathway, worsening the pathology by increasing P2Y14 mRNA expression. Pharmacological blockade of P2Y14 in eosinophils attenuated intestinal inflammation, suggesting a promising intervention for eosinophil-driven colitis [58]. Still regarding eosinophils, Karcz et al. (2021) demonstrated that P2Y14 knockout mice were able to develop allergic sensitization. This means that the receptor was not necessary for the induction of adaptive type 2 responses, including allergic sensitization and the production of IL-5 and IL-13. However, P2Y14 played a key role during the effector phase, enhancing local inflammation and recruiting eosinophils, which amplified airway eosinophilia and hyperresponsiveness in a murine model of lung asthma [59].

Curiously, P2Y14 also demonstrated playing a significant role in the fibrotic processes. Mederacke et al. (2022) identified P2Y14’s involvement in hepatic fibrosis through the activation of hepatic stellate cells in response to hepatocyte injury and death. This activation of the receptor may be related to UDP-glucose release during cellular stress, functioning as a DAMP. In animal models, the inactivation of P2Y14 significantly reduced fibrogenesis, highlighting the receptor’s therapeutic potential also in liver diseases [60].

However, contrasting with its pro-inflammatory roles in peripheral tissues, in glioma models P2Y14 activation was associated with anti-inflammatory effects. Curet and Watters (2018) demonstrated that stimulating this receptor significantly reduced IL-6 production by GL261 glioma cells, a cytokine known to correlate with poor prognosis in glioblastoma [61]. These findings indicate that while P2Y14 antagonism may be beneficial in several inflammatory conditions, to attenuate inflammation, its activation could also exert protective effects in specific scenarios, such as within the tumor microenvironment of the central nervous system, raising important questions about the circumstances under which P2Y14 activation may be protective or harmful.

## Potentially detrimental effects of P2Y14 activation

It is important to highlight that the pro-inflammatory role of receptors of the purinergic system is not always a synonym for good prognosis. Inflammation may cause several complications, like by the excessive secretion of ROS and NET formation from neutrophils’ recruitment and infiltration [62]. These inflammatory responses impair the healing process, accentuate damage, and may also play a pro-carcinogenic role in tumor cells.

Acute respiratory distress syndromes are an example of the complication that inflammation may cause due to the participation of neutrophils. The blood vessels are a semipermeable barrier that allows a controlled transport of proteins, gases, and small molecules. During respiratory diseases, neutrophils augment the permeability of blood vessels through the release of defensins and elastases that break the extracellular matrix and damage endothelial cells. This process leads to the formation of intercellular gaps, allowing plasma proteins and fluid to leak into the alveolar space. The resulting edema and protein-rich exudate impair gas exchange and contribute to the pathogenesis of acute respiratory distress syndromes [63]. In COVID-19, for instance, the exacerbated immune response leads to excessive neutrophil infiltration, aggravating systemic inflammation and contributing to lung injury through the release of reactive oxygen species, proteolytic enzymes, and NETs, which amplify tissue damage and microvascular thrombosis [64].

Therefore, Lintzmaier et al. (2021) propose the antagonism of P2Y14 by PPTN as an important therapeutic strategy to mitigate these effects by reducing neutrophil recruitment and NET formation in COVID-19 and the associated lung inflammation. This strategy demonstrates consistent potential, since P2Y14 not only promotes neutrophil chemotaxis and activation, but also regulates hematopoietic stem cell differentiation and mobilization to the lung [62]. Once in the inflamed environment, these progenitor cells may differentiate into myeloid lineages, thus sustaining neutrophil replenishment and perpetuating inflammation [65, 66]. Moreover, by impairing P2Y14 activation, this approach could also limit the systemic supply of new inflammatory cells and contribute to alleviating the respiratory compromise observed in COVID-19 and preventing the exacerbation of the bradykinin storm, one of the main drivers of severe pulmonary complications [62].

Similarly, in other diseases like acute kidney injury, activation of the P2Y14 receptor promotes recruitment of neutrophils and monocytes to the kidney, where the released cytotoxic granules contribute to tubular damage, ischemia, and consequently kidney dysfunction [46]. In turn, Mollinedo (2019) describes how breast cancer and other different tumor cells benefit from neutrophil activation; thus, the release of factors acting on tumor cells may drive them into a more activated and aggressively spreading state [67]. For instance, NET formation secreted by neutrophils contributes to the circulating tumor cells’ adhesion to the endothelium, which facilitates their migration, while the interactions between NETs and tumor cells can also activate signaling pathways within the malignant cells, promoting their survival and growth [68, 69].

Also, along with other leukocytes, neutrophils can form an immunological microenvironment beneficial for different tumor cells’ survival and proliferation [4]. Glioblastoma cells, for instance, are extremely invasive and diffuse tumors that secrete high concentrations of TGF-β, an immunosuppressive cytokine, that reduces NK and T lymphocytes’ activity. Curiously, these tumors also secrete IL-6 and IL-8, which are cytokines that promote neutrophil migration, proliferation, and extended survival [70, 71], maintaining a pro-carcinogenic state in their surroundings. Consequently, the prognosis of glioblastoma patients with high neutrophil activity is associated with increased tumor progression and malignancy [72].

Although P2Y14 exerts a relevant role in neutrophil recruitment, its blockade by PPTN, besides impairing migration, also promoted a phenotype change in mouse neutrophil physiology, from a pro-inflammatory to an anti-inflammatory role. This effect links P2Y14 activity to the detrimental inflammatory profile exerted by neutrophils that can damage healthy peripheral cells when dysregulated (as similarly observed in myocardial ischemia/reperfusion injury) [43], but could also represent an important mechanism to regulate neutrophil infiltration in the tumor microenvironment. These evidences also highlight that the neutrophils’ role in the tumor microenvironment is dual and complex, as their phenotype can range from tumor-promoting to tumor-suppressing [73].

## P2Y14 as a target for neutrophil regulation

TGF-β secreted by tumor cells favors the immunosuppression in the tumor microenvironment and also modulates the neutrophil phenotype within the tumor region, promoting their polarization toward an anti-inflammatory N2 profile, which is generally protumor [74]. In this state, neutrophils can suppress the activity of NK cells and recruit cells with anti-inflammatory functions, such as M2 macrophages and regulatory T cells, which reduce the immune response, while they are also capable of promoting angiogenesis through the synthesis and release of matrix metalloproteinase 9. In contrast, the N1 phenotype is characterized by strong inflammatory activity, with production of ROS, TNF-α, reactive nitrogen species, and other pro-inflammatory cytokines, resulting in tumor cytotoxicity and direct killing of malignant cells, as well as promoting the recruitment of inflammatory cells such as CD8+ T lymphocytes and M1 macrophages [75].

Even though P2Y14 is not directly associated with angiogenic events, the induced modifications in components of the purinergic system in immunological cells, such as enzymes and receptors, have demonstrated to be valuable tools in the search for new treatments for diseases [76]. For instance, antagonism of purinergic receptors, such as P2Y12 by clopidogrel and A2A by istradefylline, has been shown to reduce tumor growth and progression in murine melanoma models, both in vivo and in vitro [77, 78]. In this context, P2Y14’s modulation offers promising perspectives in the control of intense inflammatory responses related to neutrophil invasion. While PPTN exhibited high efficacy in blocking P2Y14 activation, with a Half Maximal Inhibitory Concentration (IC_50_) values ranging from 1 to 100 nM [31], its poor solubility in water and limited bioavailability impair its therapeutical usage [79].

To address these limitations, Jung et al. (2020) introduced biaryl scaffolds with improved pharmacokinetic properties as alternatives for P2Y14 antagonism while retaining nanomolar affinity for the receptor [79]. More recently, HQL6 was also identified as a potent and orally bioavailable P2Y14 antagonist, with IC_50_ of 3 nM, demonstrating in vivo efficacy in ameliorating acute gouty arthritis by suppressing macrophage pyroptosis [80]. Furthermore, non-nucleotide analogues such as Compound 4 have shown rapid reversal of chronic neuropathic pain in mice, as demonstrated by Mufti et al. (2020) [81].

Therefore, antagonism of P2Y14 using recent, more selective and bioavailable compounds represents promising therapeutic and alternative strategies, potentially reducing not only inflammatory interleukins’ secretion and acute neutrophil infiltration, but also impairing the neutrophils’ unwanted detrimental effects over tumor cells’ microenvironment. However, despite these promising insights, the precise contribution of P2Y14 signaling across different tissues and pathological contexts remains to be fully elucidated in vivo. Further investigations could help to fill this gap in order to determine whether P2Y14 antagonism can effectively modulate neutrophil activity, attenuate acute/systemic inflammation, or limit tumor-promoting effects without compromising host defense mechanisms.

## Conclusion

P2Y14 plays crucial roles in several immunological conditions. Activated by UDP-glucose, P2Y14 mediates pro-inflammatory signals that trigger neutrophil migration through chemokine production by altering complex intracellular cascades of second messengers. While neutrophils are essential for the first line of defense against pathogens and tissue damage, their excessive or dysregulated activity can exacerbate inflammation, induce cell stress, or promote tumor progression when cancer is already established. Thus, modulating P2Y14 activity to regulate neutrophil chemotaxis represents a promising therapeutic strategy to mitigate detrimental pro-inflammatory responses or enhance beneficial immune functions. This perspective also highlights the need for further research to elucidate the underlying mechanisms of P2Y14 signaling, fostering its development as a potential target across diverse pathological contexts.

## Data Availability

No datasets were generated or analysed during the current study.
